# The Preservation and Reuse of Lenticules Extracted via Small Incision Lenticule Extraction (SMILE): A Narrative Review

**DOI:** 10.3390/bioengineering12040380

**Published:** 2025-04-03

**Authors:** Yaohua Zhang, Jing Li, Zhiqing Wu, Yong Li, Guoxi Wu, Shengsheng Wei

**Affiliations:** 1 Shannxi Eye Hospital, Xi’an People’s Hospital (Xi’an Fourth Hospital), Affiliated People’s Hospital of Northwest University, Xi’an 710004, China; zhangyaohua_tina@163.com (Y.Z.); lijing850205@163.com (J.L.); xaszfzly2021@163.com (Y.L.); gxwu1997@163.com (G.W.); 2 Department of Ophthalmology, Hospital of Shaanxi Normal University, Xi’an 710004, China; zhiqingwu366@126.com

**Keywords:** SMILE surgery, corneal stromal lenticules, hyperopia correction, lenticular implantation, keratoconus

## Abstract

Small-incision lenticule extraction (SMILE) is a safe and effective procedure to correct myopia and myopic astigmatism. The corneal stromal lenticules extracted from SMILE surgery have good light transmission, mechanical properties, and biocompatibility, which are suitable for the treatment of a variety of corneal diseases and can solve the problem of donor cornea shortage. At present, no single method of preserving corneal stromal lenticules has been universally accepted as ideal, as the preservation of tissue integrity, optical transmittance, cellular viability, and the potential for long-term storage remain key challenges. Current approaches include short-term preservation methods such as the use of dehydrating agents and Optisol GS, and long-term preservation strategies such as cryopreservation, hydrogel nutrient capsules, and silicone oil. Standardized storage methods can improve the use of SMILE-derived lenticules as a substitute for donor corneal tissue in clinical settings. The reuse of corneal stromal lenticules is a highly regarded research area, especially in hyperopia, presbyopia, keratoconus, and some corneal ulcerative diseases, providing new possibilities for addressing corneal tissue shortage and improving surgical outcomes. Here, we review various preservation methods and clinical applications of SMILE-extracted lenticules, highlighting their potential in addressing corneal tissue shortages and the treatment of a variety of corneal diseases.

## 1. Introduction

The cornea is the primary refractive structure of the eye, composed of six main layers: the anterior corneal epithelium, an acellular collagenous Bowman’s membrane, the corneal stroma, Dua’s layer, Descemet’s membrane, and the corneal endothelium [1,2]. The corneal stroma constitutes approximately 90% of the corneal volume [3,4]. Corneal diseases are among the leading causes of blindness and visual impairment globally, particularly in developing countries, contributing significantly to socioeconomic burdens [5]. A persistent challenge in many regions is the shortage of donor corneas [6]. Although the cornea is considered immune-privileged tissue, stromal allograft rejection can still occur. Compared to fresh tissue and traditional full-thickness or lamellar keratoplasty, intrastromal lenticules exhibit reduced immunogenicity, thereby lowering the risk of allograft rejection.

Recent advancements in refractive surgery have led to the increasing popularity of small-incision lenticule extraction (SMILE), a safe and effective procedure for myopia and myopic astigmatism [7]. SMILE utilizes a femtosecond laser to create a corneal lenticule, which is extracted through a small incision (2–5 mm) without the need for a flap. This technique potentially reduces surgical time, minimizes side effects, and enhances the accuracy of corneal ablation [8,9]. Typically, corneal stromal lenticules are discarded post-surgery. However, with the increasing number of SMILE procedures, there is a growing repository of extracted stromal lenticules that can be repurposed as biomaterials. These lenticules possess good optical transmittance, favorable mechanical properties, and excellent biocompatibility, making them suitable for treating various ocular conditions such as hyperopia, presbyopia, and keratoconus, thus addressing the issue of donor corneal shortages.

Corneal transplantation depends on the effective harvesting, storage, and transportation of corneal tissue. Therefore, the preservation of corneal stromal lenticules is crucial prior to implantation. Maintaining the normal collagen architecture and cellular vitality of the extracted stromal lenticules is essential for ensuring optimal surgical outcomes and minimizing complication risks [10]. A comprehensive analysis of the best preservation methods for corneal stromal lenticules is necessary to ensure their safety in clinical applications and to improve clinical outcomes.

This article will review the preservation methods and clinical applications of SMILE-extracted lenticules, highlighting their potential in addressing corneal tissue shortages and the treatment of a variety of corneal diseases.

## 2. Storage and Preservation Methods for Corneal Stromal Lenticules

Corneal stromal lenticules, typically extracted during SMILE surgery, have garnered increasing attention as a potential resource for corneal tissue preservation and transplantation. This paper reviews various storage and preservation techniques for corneal stromal lenticules, categorized into short-term and long-term methods. Despite extensive research, no single method has been universally accepted as ideal, as the preservation of tissue integrity, optical transmittance, cellular viability, and the potential for long-term storage, remain key challenges. Standardized storage methods can improve the use of SMILE-derived lenticules as a substitute for donor corneal tissue in clinical settings.

### 2.1. Short-Term Storage

Short-term storage typically refers to preserving lenticules for a period of up to 48 h. Several methods have been explored for this purpose. Liu et al. demonstrated that human lenticules stored in various reagents at either 4 °C or room temperature for up to 48 h maintained similar tissue clarity and structural integrity, thus offering potential protocols for safe and effective short-term storage [11].

#### 2.1.1. Dehydrating Agents

Dehydrating agents such as silica gel, glycerol, and silicone oil are commonly used for short-term preservation. Among these, silica gel has shown the best performance in maintaining optical transmittance, making it a favorable choice for short-term storage of stromal lenticules [12].

Silica gel: Silica gel works by removing water from corneal tissue and inhibiting enzymatic activities and autolysis, preserving cellular integrity. Studies have indicated that lenticules stored in silica gel exhibited significantly better transparency after four weeks compared to those stored in glycerol and silicone oil. In clinical practice, the type of silicone gel commonly utilized for the preservation of corneal materials is allochronic silica gel desiccant [12].

Glycerol: Glycerol (99% anhydrous glycerol) is a cryoprotectant widely used for long-term preservation of biological tissues, including corneas. It prevents the formation of ice crystals within cells by lowering the freezing point, thus preserving cellular integrity during freezing and thawing. Glycerol also stabilizes collagen structure without altering the native collagen ultrastructure [13]. In a study by Li et al. [14], glycerol-preserved corneas demonstrated well-maintained transparency and collagen structure after 4 weeks of storage. However, glycerol is more suitable for short-term preservation at room temperature or 4 °C rather than prolonged storage.

Silicone oil: Silicone oil, typically used as an internal tamponade agent in ophthalmic surgeries, has shown promise in preserving corneal tissues. It is stable, exhibits low toxicity, and has higher hydrophobicity than glycerol, making it an attractive option for tissue preservation. However, long-term storage with silicone oil has shown a reduction in optical transmittance after extended periods [12].

#### 2.1.2. Optisol GS and Other Preservation Solutions

Optisol GS: Optisol is a medium used for midterm corneal storage, typically up to 14 days. It effectively preserves corneal tissue integrity and endothelial cell viability, allowing for safe transport of donor corneas over long distances [15,16]. However, the high cost of Optisol limits its broader application in lenticule storage. Studies have shown that Optisol maintains transparency and viability for up to two weeks at 4 °C, making it an ideal medium for temporary preservation under emergency conditions [17].

Phosphate-Buffered Solution (PBS): PBS is commonly used for short-term storage (up to 48 h) of corneal tissue. PBS is an isotonic solution that maintains tissue hydration without affecting cellular integrity, although it does not offer the same level of protection as glycerol or Optisol for longer storage periods.

Dulbecco’s Modified Eagle’s Medium (DMEM): DMEM supplemented with fetal bovine serum (FBS) has been used for in vitro culture of corneal endothelial cells. While DMEM is effective for short-term storage and cell culture, its application for lenticule storage is limited, as it requires careful management of temperature and serum concentrations [18].

### 2.2. Long-Term Storage

Long-term storage of corneal stromal lenticules generally refers to preservation for extended periods, ranging from several weeks to months or even years. Techniques for long-term storage include cryopreservation, hydrogel nutrient capsules, and advanced dehydrating agents.

#### 2.2.1. Cryopreservation

Cryopreservation is one of the most widely used methods for long-term preservation of biological tissues, including corneal lenticules. The principle behind cryopreservation is the rapid cooling of tissue to very low temperatures to reduce metabolic rates and enzymatic activity, thereby maintaining cellular viability and tissue functionality. Cryopreservation allows tissues to be stored in liquid nitrogen at temperatures between −80 °C and −196 °C, with minimal risk of cellular damage [19,20].

Advantages and Challenges: Cryopreserved lenticules maintain their structural integrity and collagen architecture, with no significant difference in wound healing outcomes when compared to fresh lenticules [21]. However, cryopreservation is not without its drawbacks. The freezing and thawing processes can induce cryoinjury, leading to cell death and disruption of collagen fibers [22,23]. Despite this, cryopreservation remains a viable method for long-term lenticule storage, especially when it comes to allogeneic transplantation, where immune rejection and epithelial ingrowth are not typically a concern [10,21,24].

Cryopreservation Protocols: Cryopreservation protocols involve meticulous procedures to balance tissue viability and structural preservation. Standardized protocols typically begin with tissue processing in sterile environments using laminar flow hoods to minimize contamination risks [21]. Lenticules are washed in antibiotic/antimycotic solutions and transferred into cryovials containing cryoprotective media. A critical step involves the gradual addition of cryoprotectants such as dimethyl sulfoxide (DMSO) or glycerol to achieve final concentrations (e.g., 10% DMSO in 10% serum-containing medium), which mitigate ice crystal formation during freezing [10,21].

The freezing process requires specialized equipment to ensure controlled cooling rates. Studies have employed programmable freezing devices or passive cooling containers like the “Mr. Frosty” system (Thermo Fisher Scientific, Roskilde, Denmark), which maintains a cooling rate of approximately −1 °C/min when placed in a −80 °C freezer overnight before transferring specimens to liquid nitrogen [10]. Alternatively, direct liquid nitrogen vapor-phase freezing protocols achieve ultralow temperatures (−196 °C) using canister-based storage systems (e.g., IBP containers, Indian Oil Corporation Limited) [21]. Liquid nitrogen storage has demonstrated efficacy in preserving lenticular structural integrity and cellular viability for ≥1 month [10], with potential for extended preservation under stable conditions.

Despite these advances, clinical adoption remains limited by technical complexities. Challenges include the need for continuous liquid nitrogen replenishment, high costs of automated freezing equipment, and risks of cryoinjury during freeze–thaw cycles. These barriers disproportionately affect resource-constrained settings, underscoring the need for simplified protocols compatible with standard laboratory infrastructure [10,21].

#### 2.2.2. Hydrogel Nutrient Capsules

A novel approach for long-term preservation of corneal stromal lenticules involves the use of hydrogel nutrient capsules. These capsules, made from natural polysaccharides such as sodium alginate and chondroitin sulfate, simulate the human tear film environment, providing optimal conditions for lenticule preservation. The use of nutrient capsules has been shown to maintain the optical transmittance, structural integrity, and cellular activity of corneal lenticules for extended periods—up to one year [25,26].

Benefits: The primary advantage of nutrient capsules is their ability to preserve lenticules without the adverse effects associated with traditional preservation methods like cryopreservation. The encapsulation method maintains tear film homeostasis, ensuring that the corneal tissue remains viable over long periods [26]. This approach also minimizes the risk of cellular dehydration or damage, making it an ideal solution for long-term storage and transplantation.

#### 2.2.3. Silicone Oil

In addition to being used for intraocular tamponade in surgeries, silicone oil has potential as a storage medium for corneal stromal lenticules. Its low toxicity and high stability suggest that it could be a promising option for long-term preservation. Silicone oil’s hydrophobic properties help maintain tissue integrity by preventing water loss and preserving the collagen structure [12].

### 2.3. Preservation Challenges and Future Directions

While several preservation methods for corneal stromal lenticules have been explored, challenges remain, particularly with regard to achieving long-term storage without compromising tissue integrity. Maintaining optical clarity, preserving collagen ultrastructure, and ensuring cellular viability are essential criteria for successful lenticule preservation.

Future research should focus on optimizing the cryopreservation process, exploring new cryoprotectants, and improving the formulation of nutrient capsules. Additionally, developing cost-effective methods for long-term preservation, such as low-cost glycerol storage or improving the performance of silica gel desiccants, will be crucial for broader clinical applications, particularly in resource-limited settings.

## 3. Re-Utilization of Corneal Stromal Lenticules

### 3.1. Lenticule Intrastromal Keratoplasty (LIKE)

LIKE is an innovative surgical technique that utilizes lenticules extracted through SMILE surgery for the treatment of various ocular conditions, including hyperopia, presbyopia, keratoconus, and post-LASIK ectasia. Because the implanted lenticule is placed in the corneal stroma and isolated from the limbal lymphatic vessels, cytokines, and immune cells in the tear fluid and aqueous humor, it may have reduced exposure to immune-related factors, potentially lowering the risk of immune-mediated rejection [27]. This approach capitalizes on the natural properties of corneal tissue, offering potential advantages over synthetic alternatives, including improved biocompatibility and reduced risk of complications.

#### 3.1.1. Hyperopia Correction

Hyperopia, a common refractive error in both children and adults, occurs when parallel light entering the eye is focused behind the retina, resulting in a blurred image. It can be corrected using spectacles, contact lenses, or surgical methods such as laser in situ keratomileusis (LASIK) and photorefractive keratectomy (PRK). However, these surgical options are often limited in patients with higher degrees of hyperopia (≥+5.00 diopters [D]), who experience higher rates of complications, including hyperopic regression and higher-order aberrations (HOAs) [28,29].

Recent studies have explored the use of corneal stromal lenticules as a potential alternative for hyperopia correction. Pradhan et al. [30] were pioneers in implanting SMILE lenticules from myopic patients into hyperopic recipients, resulting in decreased spherical equivalents and improved corneal topography. However, subsequent studies indicated an undercorrection for high hyperopic treatments using this method [21]. The observed posterior corneal changes were identified as a significant factor contributing to this undercorrection, a phenomenon predicted by finite element modeling [31].

Zhang et al. [32] studied the outcomes of patients with hyperopic astigmatism treated with SMILE surgery combined with allogeneic intrastromal lenticule inlay. The results showed that SMILE combined with intrastromal lenticule inlay can be used to correct high hyperopia with astigmatism, effectively improving the visual effect of patients.

Wu et al. [33] and Moshirfar et al. [34] investigated the implantation of allogeneic refractive lenticules to correct moderate-to-high hyperopia. Ganesh et al. [21] demonstrated the feasibility of cryopreserving extracted corneal lenticules after SMILE surgery for potential use in human subjects. Additionally, Sun et al. [35] reported on the safety and predictability of implanting autologous lenticules obtained through SMILE for hyperopia correction. Li et al. [36] studied the re-innervation of implanted lenticules and microscopic morphological changes in the corneal architecture of the recipient cornea in five patients (with myopia in one eye and hyperopia in the contralateral eye) who received SMILE in the myopic eye and femtosecond laser in situ keratomileusis (FS-LASIK) combined with lenticule implantation in the contralateral hyperopic eye by confocal microscopy in vivo. Preliminary findings suggest that nerve fibers will regenerate into the implanted lenticule after autologous lenticule implantation.

Lenticular implantation presents an attractive alternative solution, potentially decreasing the risk of corneal instability while increasing the ability to correct higher levels of hyperopia. Synthetic corneal implants, while available, are associated with significant risks, such as chronic inflammation, nonepithelialization, corneal melt, glaucoma, and endophthalmitis, which limit their clinical utility [37]. In contrast, allogeneic implants demonstrate high bioavailability, translating to a reduced risk of complications.

Ganesh et al. [21] utilized lenticules from SMILE surgeries to address hyperopia, reporting significant postoperative visual acuity improvements without rejection during follow-up. These findings suggest that refractive lenticules extracted after SMILE are both safe and effective for human use. However, SMILE lenticule transplantation shows good predictability for moderate hyperopia; it demonstrates poorer predictability for high hyperopia without lens intervention.

Moshirfar et al. [34] reported the first case of allogeneic SMILE lenticule implantation for hyperopia correction in the United States, indicating its effectiveness based on postoperative visual outcomes. The myopic correction algorithm for SMILE produces positive meniscus lenticules, which are thicker in the center and gradually thinner toward the periphery. Implanting these lenticules induces an increase in anterior corneal curvature, effectively correcting hyperopia in humans. Most eyes achieved a postoperative spherical equivalent (SE) within 0.50 D of the target, although slight steepening of the posterior corneal curvature and a predicted doughnut pattern in epithelial thickness were observed.

Two primary factors contribute to the undercorrection of hyperopia: changes in the posterior surface and epithelial thickness remodeling [30]. The diameter of the implanted lenticule may significantly impact regression due to epithelial remodeling, yet it is constrained by the donor corneal pachymetry and the myopic refraction to be corrected. Unlike myopia, correcting hyperopia requires creating a furrow-like ring zone in the cornea’s paracentral region. Both PRK and LASIK are subtractive procedures that steepen the cornea by ablating midperipheral tissue, resulting in an abnormal hyperprolate corneal shape associated with significant regression, particularly in higher degrees of refractive errors [38,39].

In the myopic SMILE procedure, the extracted lenticule is convex-shaped, designed to flatten the central cornea. By implanting the central portion of this convex-shaped lenticule, the corneal anterior curvature can theoretically be reshaped to be more hyperprolate, enhancing near and intermediate vision [40]. Biological corneal inlay implantation represents a tissue additive procedure with potential advantages over corneal stromal laser ablation in terms of reversibility [41].

**Figure 1:**
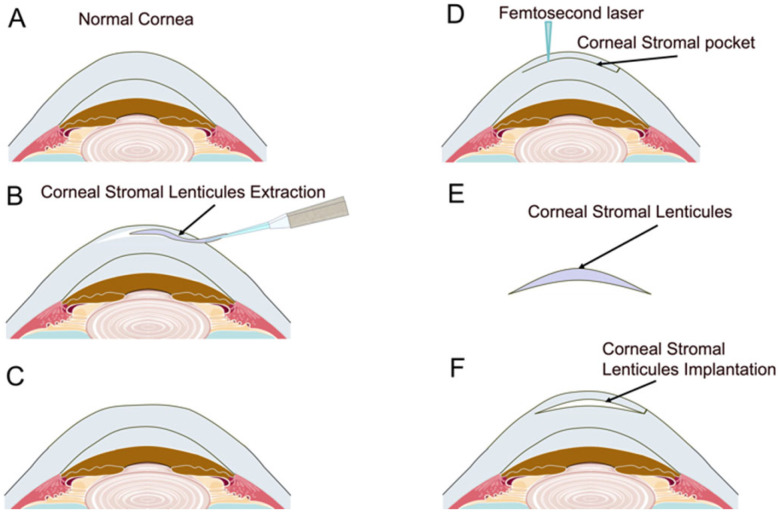
In the normal cornea of myopic astigmatism patients (**A**), femtosecond laser is used to scan the anterior corneal stroma to form a corneal stromal lenticule. After removing the corneal stromal lenticule (**B**), the curvature of the anterior corneal surface becomes flatter (**C**), which can be used to correct myopic astigmatism. At the same time, femtosecond laser can be used to create a stromal pocket in the anterior corneal stroma of hyperopic astigmatism patients (**D**). The corneal stromal lenticule with a thick central part and thin peripheral part (**E**), which is removed from myopic astigmatism patients, is implanted into the stromal pocket of hyperopic astigmatism patients, making the curvature of the central area of the anterior surface of their cornea steeper (**F**). This can be used to correct the refractive power of hyperopic astigmatism patients.

Research indicates that implanting a lenticule derived from SMILE effectively results in central steepening of the cornea, central hyperprolate changes, reasonable anterior surface elevation, and acceptable predictability [42], which may provide a potentially valuable method for correcting moderate-to-high hyperopia. The decellularization process holds promise for reducing the occurrence of stromal rejection while maintaining treatment efficacy (Figure 1).

#### 3.1.2. Presbyopia Treatment

Presbyopia, characterized by a gradual decline in the eye’s focusing ability with age, is prevalent among individuals over 40 years old [43,44]. Current correction strategies include spectacles, contact lenses, surgical interventions, and pharmaceutical treatments. Among these, synthetic and biological corneal inlays have gained traction as effective modalities for presbyopia correction [38,45].

The use of corneal stromal volume restoration through SMILE lenticule re-implantation offers an opportunity to restore the non-dominant eye to a previous low myopia state, facilitating a monovision approach. Unlike synthetic inlays, which can lead to complications such as inflammatory responses and interference with corneal diffusion processes [46,47], biological inlays derived from allogenic tissue provide better integration and reduced inflammatory risks.

Jacob et al. [40] demonstrated the feasibility of using allogeneic presbyopia inlays, showing that extracted SMILE lenticules can be trephined to a 1 mm diameter and implanted under a cap at a depth of 120 μm. This technique effectively increases the central radius of curvature, yielding a hyperprolate corneal shape that stabilizes near vision. The use of allogenic tissue enhances biocompatibility and integration, thereby reducing the risk of corneal necrosis and melt.

#### 3.1.3. Keratoconus and Corneal Ectasia

Keratoconus, a prevalent form of corneal ectasia, is characterized by progressive thinning and bulging of the cornea, leading to visual impairment due to irregular astigmatism and central scarring [48,49]. Treatment options for keratoconus vary based on severity and progression and include glasses, contact lenses, corneal collagen crosslinking, intrastromal corneal ring implantation, and keratoplasty. In severe cases, penetrating keratoplasty (PKP) or lamellar keratoplasty may be necessary to improve visual acuity [50,51,52]. However, this method is limited by relatively high rates of immune intolerance, slow postoperative recovery, high costs, and a shortage of donor corneas [53].

**Figure 2:**
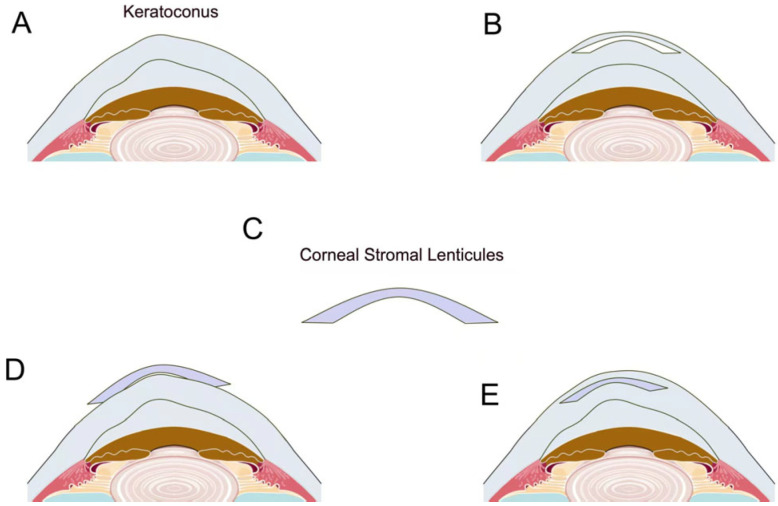
The local corneal thickness of keratoconus becomes thinner, and the anterior surface curvature increases, resulting in highly irregular myopic astigmatism (**A**). The high myopic astigmatism in patients with keratoconus can be corrected using the concave lenticules removed from hyperopic SMILE surgery (**B**). This concave lenticules extracted are biocompatible, transparent stromal tissues with a concave geometry, enabling their potential use in correcting refractive errors (**C**). Method 1 is used to cover the surface of keratoconus where the corneal epithelium has been removed, also known as surface lenticules surgery (**D**). Method 2 is when stromal lenticules can be implanted into the corneal stroma using a femtosecond laser to create a pouch, also known as stromal lenticule implantation (**E**).

Lenticular implantation is emerging as a viable option for treating keratoconus. The hyperopic algorithm for SMILE creates negative meniscus-shaped lenticules, which are thicker at the periphery and thinner at the center [54,55]. These lenticules can be implanted into the stroma, resulting in central corneal flattening and increased stromal thickness [56], which is beneficial for keratoconus management [57] (Figure 2). However, the extent of refractive correction by lenticule implantation cannot be accurately predicted due to the postoperative tissue remodeling process and the edema of the lenticule. Contrary to concave lenticules, convex or positive meniscus lenticules add more thickness to the central cornea to strengthen the expanding cone at the center and control the progression of keratoconus.

In 2015, Ganesh et al. [58] reported a method of combining femtosecond intrastromal lenticular implantation and accelerated collagen cross-linking for treating keratoconus. Their study indicated that this combination effectively stabilizes keratoconus while improving corneal optics and reducing aberrations. The use of customized necklace SMILE implants aimed to increase corneal thickness, reduce keratometric values, and enhance corneal regularity in cases of inferior steepening.

Jadidi et al. [59] utilized femtosecond laser technology to create a desirable corneal lenticule with precise diameter, depth, and shape, as well as an intra-stromal pocket, in keratoconus patients. The shape of the lenticule is determined according to the type of keratoconus; the central keratoconus is circular, the inferior keratoconus is crescent shaped, and the asymmetric bow-tie shaped keratoconus is round with a size adjusted for mesopic pupil size. The depth of the pocket is set to 250 microns of the corneal thickness at the incision site. According to their report, all patients showed significant improvement in corrected distance visual acuity, and this technique appears to be a feasible and safe treatment option for keratoconus.

Pedrotti et al. [60] used an excimer laser to thin the ex vivo cornea to a thickness of 200 μ m and then inflated it to 100 mmHg to create an ex vivo ectasia model (recipient corneas). After using femtosecond laser to fabricate biconvex stromal lenticules and placing them into an intrastromal pocket inside the ectatic recipient corneas, the thickness of the recipient cornea significantly increased, while the maximal posterior elevation from the best-fitted toric ellipsoid significantly reduced. Mastropasqua et al. [61] highlighted the successful application of stromal lenticule addition keratoplasty for advanced keratoconus, demonstrating that this technique can stabilize the cornea and improve visual outcomes. The addition of lenticular tissue in the mid-periphery around the cone induces relative flattening in the center, modifying the corneal shape to a more natural prolate configuration.

Jin et al. [62] compared the efficacy of small-incision femtosecond laser-assisted corneal concave lenticule implantation (SFII) and penetrating keratoplasty (PKP) in patients with progressive keratoconus. The results showed that both surgeries could stabilize corneal volume and improve visual acuity. Compared with the PKP group, SFII surgery was less invasive and more efficient.

Doroodgar et al. [63] evaluated the effectiveness of implanting customized necklaces and ring 120° forms lenticules obtained from myopic SMILE surgery into 22 patients with advanced keratoconus. The results showed that the corneal density, thickness, keratometry, and vision of all cases were significantly improved. Stromal lenticule addition keratoplasty produces transitory nerve plexus density reduction and minor inflammatory reaction that rapidly decreases during the first month. There was no sign of stromal opacification or stromal rejection in 1 year of follow-up.

Riau et al. [64] reviewed and performed a meta-analysis of the clinical outcomes of the femtosecond laser-assisted stromal keratophakia for keratoconus. They found that various factors, including the shape of lenticules, whether to crosslink or not, and implantation depth, may be related to visual improvement. Concave-shaped lenticule implantation seems to improve corneal curvature and refractive power more in patients with advanced keratoconus. The cross-linking of lenticules can stabilize the refractive status of the corneas earlier and reduce refractive regression over time. The depth of implantation is also crucial, as implantation at a 110 μm depth resulted in 66–78% of the targeted refractive correction. In contrast, implantation of lenticule at 160 μm depth resulted in only 42–50% of the targeted correction.

Fan et al. [65] used lenticules obtained through SMILE surgery as a source of human tissue to evaluate the effectiveness of corneal cross-linking (CXL). The results showed that the CXL effect did not vary significantly with protocols using 3–18 mW/cm^2^ irradiance, but there was a significant efficacy drop with 30 mW/cm^2^ irradiance.

#### 3.1.4. Post-LASIK Ectasia

Tan et al. [66] first proposed using lenticules for treating post-LASIK ectasia, indicating their potential as a viable option for patients experiencing corneal ectasia following LASIK surgery. Subsequent studies, including one by Jiang et al. [67], introduced a novel method of lamellar keratoplasty in which the SMILE-extracted lenticules were placed in the cone region between the LASIK corneal flap and the stromal bed after gently lifting the flap. Then, they placed the LASIK corneal flap back in its previous position and used the SMILE-extracted lenticules to address post-LASIK ectasia. Li et al. [68] successfully treated patients with post-LASIK corneal ectasia using SMILE-extracted lenticules. Li et al. [69] also studied the morphological changes in the cornea and implanted lenticules in eight eyes with corneal ectasia after 3 years of laser in situ keratomileusis (LASIK) and implantation using a lenticule from small-incision lenticule extraction (SMILE). The study demonstrated that keratocyte numbers and morphology gradually recovered, with recipient keratocytes migrating into the stroma and potential regeneration of the sub-basal nerve plexus occurring at the lenticule interfaces.

The combination of tissue addition and accelerated collagen cross-linking presents a feasible option for managing low-to-moderate keratoconus [58]. The primary goals of keratoconus treatment are to stabilize the cornea and improve its optical quality by reducing aberrations. By adding corneal tissue in the mid-periphery, the technique facilitates relative flattening in the center and modifies the corneal shape.

These studies showed that tuck-in lamellar keratoplasty using SMILE-extracted lenticule might be an effective alternative to conventional treatment for severe cases of post-LASIK ectasia with thin cornea.

### 3.2. Corneal Patch Grafting

Corneal patch grafting has emerged as a significant technique in ophthalmic surgery, particularly for managing corneal perforations, dystrophies, and other surface irregularities. This method utilizes lenticules obtained from SMILE procedures, leveraging their acellular properties to mitigate rejection risks and enhance healing outcomes. The applications of corneal patch grafting are mainly focused on the treatment of corneal perforations, dystrophies, and complications arising from various ocular conditions.

#### 3.2.1. Corneal Ulcer and Perforation

Corneal perforation is a critical condition that can arise from various corneal diseases, including ulcers and dystrophies [70]. Corneal ulcers and perforations typically present poor prognoses, necessitating prompt surgical intervention to prevent severe complications such as profound visual loss and intraocular infections [71,72]. Tectonic keratoplasty, whether lamellar or penetrating, is often recommended for extensive corneal lesions. PKP is considered the gold standard for extensive corneal lesions [73]. However, the demand for donor corneas often exceeds supply, particularly in developing countries, making alternative solutions essential. The use of SMILE-derived lenticules offers a promising solution for corneal ulcers and perforations. Their clear, high-quality tissue provides a viable option for patch grafting, with studies indicating that lenticules can effectively preserve corneal integrity and reduce inflammatory responses [74]. The technique involves the careful removal of necrotic tissue, followed by the application of lenticules shaped to fit the defect.

Bhandari et al. [75] explored the application of SMILE-derived glued lenticule patch grafts in microperforations and partial-thickness corneal defects, demonstrating their effectiveness in preserving corneal integrity and facilitating healing without complications. Furthermore, the use of fibrin glue to secure the lenticule enhances adhesion and stability during the healing process. The acellular nature of lenticules reduces the risk of immune rejection, eliminating the need for steroid drops or immune inhibitors. This characteristic is particularly advantageous in emergency situations where donor tissue may not be readily available.

**Figure 3:**
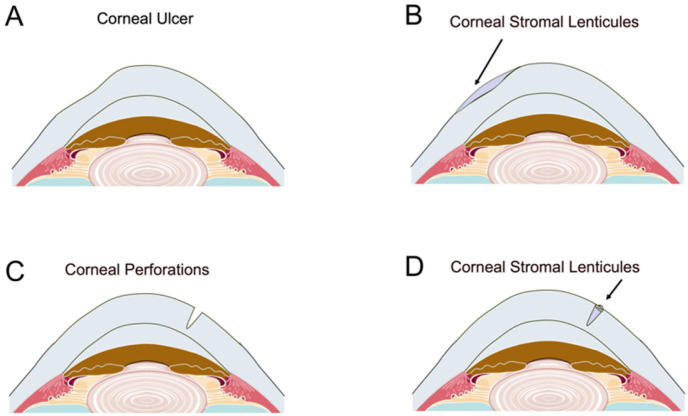
The application of SMILE-derived glued lenticule patch grafts in corneal ulcers and perforations. (**A**,**B**): a single layer of SMILE-extracted lenticule was used in corneal ulcers; (**C**,**D**): crimped stromal lenticule was applied to corneal perforations.

Yang et al. [74] reported a novel tectonic keratoplasty technique utilizing SMILE-extracted lenticules for corneal ulcers and perforations. In their study, necrotic tissue was removed, and lenticules were shaped to fit the lesion, effectively sealing the perforations without complications such as graft displacement or aqueous leakage (Figure 3).

Abd Elaziz et al. [76] observed similar success in a cohort of seven patients treated with lamellar keratoplasty using a single layer of SMILE-extracted lenticules, augmented with an amniotic membrane to enhance healing. The combination of these techniques allows for effective closure of corneal perforations while providing nourishment and growth factors essential for recovery.

#### 3.2.2. Corneal Dystrophies and Epikeratophakia

In addition to perforations, corneal dystrophies present another challenge in ophthalmic surgery. Zhao et al. [77] investigated the use of donor lenticules obtained through SMILE in an epikeratophakia technique combined with phototherapeutic keratectomy (PTK). This innovative approach provides an alternative for patients with corneal dystrophies, particularly those with thin corneas who may not be suitable candidates for penetrating keratoplasty.

The combination of PTK and epikeratophakia using SMILE-derived lenticules offers a viable option for treating corneal dystrophies while minimizing the risks associated with more invasive procedures. The lenticules provide a scaffold for corneal epithelial regeneration, promoting healing while maintaining corneal transparency and integrity.

#### 3.2.3. Applications in Other Ocular Conditions

Corneal lenticules have also been utilized in various other ocular conditions, including limbal dermoids, Mooren’s ulcer, and pterygium. Limbal dermoids, common in children, can be surgically managed using lenticule keratoplasty, which retains globe integrity and minimizes complications such as postoperative limbal ectasia [78]. Jacob et al. [79] reported successful cosmetic and refractive outcomes using sutureless fibrin glue-assisted tectonic grafts with SMILE-extracted lenticules in superficial limbal dermoid cases. Wan et al. [78] reported the successful results of using SMILE-extracted lenticules to assist in corneal patch graft for the treatment of superficial limbal dermoid. All three patients recovered well, and in the final follow-up, there was an improvement trend in visual acuity and astigmatism in all eyes.

Li et al. [80] observed five patients with corneal dermoids who underwent corneal dermoid excision combined with fibrin glue (FG) boned multi-layer corneal lenticules transplantation treatment. The results showed that all patients were satisfied with the postoperative cosmetic effect, and the lenticule grafts grew well without rejection, maintaining transparency during follow-up.

Pterygium, a fibrovascular tissue growth on the ocular surface, may require surgical excision. Recent studies suggest that SMILE-derived lenticules can serve as a novel graft source for pterygium surgery, providing a transparent and biocompatible option for grafting [81]. Pant et al. [82] reported that in the case of recurrent pterygium complicated with thin cornea, the use of SMILE-extracted lenticules for lamellar keratoplasty showed promising results, offering an effective alternative to traditional grafting techniques. Jacob et al. [83] also used SMILE extracted lenticules with fibrin glue as patch grafts to successfully treat multiple corneal macroperforation cases with complicated pseudopterygium excision with deep intracorneal extension.

Hu et al. [84] evaluated the clinical efficacy of PTK combined with fibrin glue fixation of corneal stromal lenticules extracted from SMILE for the treatment of superficial corneal opacities after one year. The results have shown that this method can improve long-term visual acuity and is a new, safe, and effective treatment for treating superficial corneal opacities.

#### 3.2.4. Glaucoma Drainage Implantation Surgery

The use of corneal lenticules extends to glaucoma drainage implantation surgery, where they serve as patch grafts to prevent tube exposure. Exposure of drainage tubes can lead to severe complications, including endophthalmitis [85]. Spierer et al. [86] demonstrated that partial thickness corneal tissue from SMILE can effectively cover the anterior part of the tube shunt, improving cosmetic outcomes and reducing the risk of complications. Wang et al. [87] compared the effect of different SMILE lenticule thicknesses on the tube exposure and surgical success rate in glaucoma drainage implant surgery, and they identified optimal corneal graft thickness for patch grafts in glaucoma drainage surgeries, noting that two layers of lamellar corneal tissue (240–300 μm) effectively reduced tube exposure and rejection rates. In some unique circumstances, three layers of lamellar corneal tissue (approximately 450 μm) may be required; however, thick grafts may enhance friction against the eyelids and cause discomfort. And it can lead to instability of the tear film, resulting in a concave cornea. In addition, one donor can only provide two corneal slices, and three layers of tissue come from two donors, which increases the risk of rejection for the recipient.

Wang et al. [88] compared the efficacy of using three-layer lenticules extracted from SMILE and the sclera as patch grafts to prevent exposure of glaucoma drainage tubes. There was no statistically significant difference in the incidence of surgical complications such as graft thinning and conjunctival melting between the two groups. But the cornea slice acquired from SMILE surgery is easy to acquire, safe, and cheaper, providing patients with a better cosmetic appearance.

Another study showed that decellularized lenticules significantly increased bleb survival and decreased intraocular pressure postoperatively in glaucoma filtration surgery on a rabbit model by acting as a physical adhesion barrier [89].

#### 3.2.5. Regenerative Corneal Engineering

The advent of regenerative corneal engineering has opened new avenues for utilizing SMILE-derived lenticules. These lenticules serve as accessible sources of collagen-rich extracellular matrix (ECM) scaffolds, exhibiting high mechanical strength, biocompatibility, and transparency. Decellularization techniques enable the removal of cellular components while preserving the structural integrity of the matrix, reducing the risk of host immune rejection [90,91,92].

Recent studies have demonstrated the potential of decellularized lenticules in promoting the differentiation of mesenchymal stem cells into corneal epithelial cells [93]. This innovative approach offers a promising therapeutic modality for corneal tissue engineering, utilizing patient-derived induced pluripotent stem cells (iPSCs) seeded on decellularized lenticules to generate coherent stratified squamous epithelial sheets.

Hong et al. [94] integrated decellularized corneal lenticules into compressed collagen to form a sandwich structure of biocomposite material, in order to create a suturable limbal epithelial stem cell (LESC) carrier for the treatment of limbal stem cell deficiency (LSCD). The clinical efficacy of LSCD in ocular surface reconstruction was confirmed in the eyes of an LSCD rabbit model. Surovtseva et al. [95] found that corneal fibroblasts reversed into keratocytes (rCF) in ReLEx SMILE lenticules could promote the recovery of transparent corneal stroma in a mouse model of mechanical corneal injury.

#### 3.2.6. Ocular Drug Delivery System

Decellularized human stromal lenticules present a promising biocompatible and non-immunogenic natural scaffold for potential ocular drug delivery applications [96]. This system enables the achievement of effective drug concentrations in the eye over a sustained period. Corneal stromal lenticules serve as a viable bio-scaffold, maintaining transparency and native stromal architecture. The cornea, known for its immune privilege, particularly in the corneal stroma due to the low immunogenicity of keratocytes, enhances the biocompatibility of this drug delivery system [97].

## 4. Conclusions and Perspectives

The clinical application of human corneal stromal lenticules in corneal diseases requires extensive foundational research to assess its safety and efficacy. Previous studies have utilized Raman spectroscopy to analyze the composition of the corneal stromal lens, revealing key components such as proline, collagen, and lipids, aiding in a deeper understanding of the biochemical characteristics of corneal tissue [98]. Furthermore, research on the effects of femtosecond laser on corneal tissue provides important insights into early damage patterns and tissue changes post-surgical intervention, contributing to the enhancement of surgical techniques and mitigation of potential risks [99]. Additionally, the analysis of corneal stromal lenticules provides crucial data for studying metabolic biomarkers and proteomics in myopia. Identification of potential biomarkers such as decanoic acid and arginine–proline, along with understanding changes in protein expression, enables a more profound comprehension of the pathogenesis of myopia, offering new directions for disease diagnosis and treatment [100]. Moreover, research on corneal stromal lens involves exploration of tissue histology and biomechanical properties. Advanced techniques like spectral domain optical coherence tomography allow for observation of histological changes in myopic corneal tissue, while elucidating the role of important proteins such as CRY-λ1 [101,102]. Investigation into the biomechanical properties of the cornea aids in evaluating structural alterations and provides a basis for the safety and efficacy of refractive surgeries.

In conclusion, the role of human corneal stromal lenticules in foundational research is multifaceted, ranging from unveiling composition and biochemical characteristics to exploring tissue histology, biomechanical properties, and disease mechanisms, offering crucial support and guidance for research and the clinical treatment of corneal diseases. To further promote and apply stromal lenticules derived from SMILE, larger-scale, multicenter clinical trials are needed to validate its safety. Extensive in vitro and in vivo studies have demonstrated its feasibility and safety; however, further foundational and clinical research is necessary to comprehensively understand its underlying mechanisms and advance clinical applications.

## Data Availability

The datasets generated and/or analyzed during the current study are available from the corresponding author, Sheng-Sheng Wei (rsdrwss001@163.com), upon reasonable request.
